# Complete PSA Response Following Stereotactic Ablative Radiotherapy for a Bony Metastasis in the Setting of Castrate-Resistant Prostate Cancer

**DOI:** 10.7759/cureus.365

**Published:** 2015-10-26

**Authors:** Jelena Lukovic, George Rodrigues

**Affiliations:** 1 Department of Oncology, Department of Radiation Oncology, London Regional Cancer Program, London, Ontario, CA; Schulich School of Medicine & Dentistry, Western University, London, Ontario, CA; 2 Department of Radiation Oncology, London Regional Cancer Program, London, Ontario, CA; Schulich School of Medicine & Dentistry, Western University, London, Ontario, CA

**Keywords:** stereotactic body radiotherapy, oligometastases, castrate-resistant prostate cancer

## Abstract

A majority of patients with castrate-resistant prostate cancer ultimately develop distant metastases, with bone being the most common site of spread. Classically, systemic therapy has been considered the standard of care for patients with metastatic cancer. Emerging evidence, however, suggests that an intermediate oligometastatic state, between limited disease and widespread metastases, exists; theoretically, with locally ablative treatment, patients may be curable. We describe a complete PSA response following aggressive management, using stereotactic body radiotherapy (SBRT), of an oligometastatic spine lesion in the setting of castrate-resistant prostate cancer (CRPC). This case report supports the use of SBRT in oligometastatic CRPC and suggests that management of limited metastases may provide good long-term outcomes in well-selected patients.

## Introduction

A majority of patients with castrate-resistant prostate cancer ultimately develop distant metastases, with bone being the most common site of spread. Classically, systemic therapy has been considered the standard of care for patients with metastatic cancer. Emerging evidence, however, suggests that an intermediate oligometastatic state, between limited disease and widespread metastases, exists; theoretically, with locally ablative treatment, patients may be curable. In this case report, we describe a complete PSA response following aggressive management of an oligometastatic lesion in the setting of castrate-resistant prostate cancer.

## Case presentation

**Figure 1:**
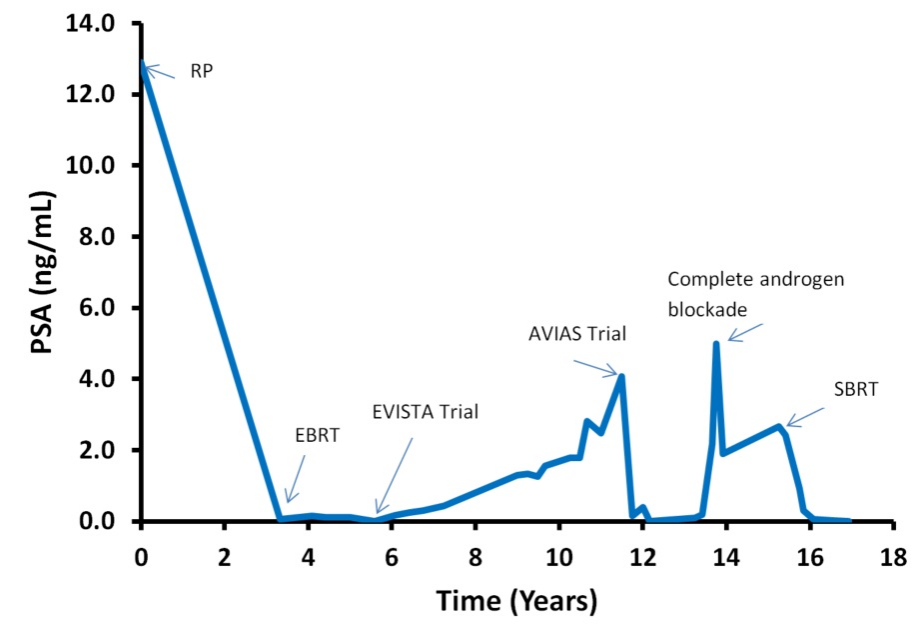
Mr. X’s PSA trend throughout treatment. RP – Radical prostatectomy, EBRT – External beam radiotherapy, SBRT – Stereotactic body radiotherapy.

In 1998, Mr. X underwent a radical retropubic prostatectomy and bilateral lymph node dissection for an intermediate risk prostate adenocarcinoma. His preoperative PSA was elevated at 12.9 and final pathology revealed Gleason 7 (3+4) disease and no evidence of extracapsular extension, no seminal vesicle invasion, no lymphovascular invasion, and nine negative lymph nodes. His postoperative PSA was undetectable. He underwent salvage radiotherapy to a dose of 7000 cGy in 35 fractions in 2001 for a PSA rise to 0.23. His PSA continued to rise post-radiotherapy, and he was enrolled in the EVISTA trial and started on gefitinib [1]. The primary objective of this trial was to evaluate the efficacy of gefitinib in patients with early biochemical failure post-prostatectomy. Mr. X tolerated the medication reasonably well with his primary side effect being a forearm rash. His PSA trend and response throughout his clinical course is shown in Figure 1.

In 2009, when Mr. X’s PSA rose to 4.08, he was taken off the EVISTA trial and started on the AVIAS trial, which randomized patients to androgen blockade followed by dutasteride, 0.5 mg daily, vs. placebo to assess whether the length of the off treatment interval in men receiving intermittent androgen deprivation therapy for localized prostate cancer could be increased [2]. The trial was blinded, and it is unknown which arm Mr. X was randomized to. On hormone therapy, Mr. X experienced fatigue, lethargy, persistent hot flashes, and deteriorating erectile function. In October 2011, with a PSA of 5, he was removed from the study and was started on a complete androgen blockade.

Mr. X’s PSA continued to rise despite complete androgen blockade and reached 2.43 in April 2013. An MRI performed during that time to assess unrelated neurologic complaints incidentally revealed a marrow signal change in the T10 vertebral body, primarily on the left side. This was confirmed with a bone scan, which demonstrated an area of intense uptake in the T10 left hemivertebra. No other areas of focal uptake were noted. Mr. X was completely asymptomatic from this lesion with no thoracic back pain, no extremity weakness, no perineal numbness, and no paresthesias. Restaging investigations that were done, including CT of the chest, abdomen, pelvis, and spine, redemonstrated the T10 sclerotic metastasis and no other sites of disease.

**Figure 2:**
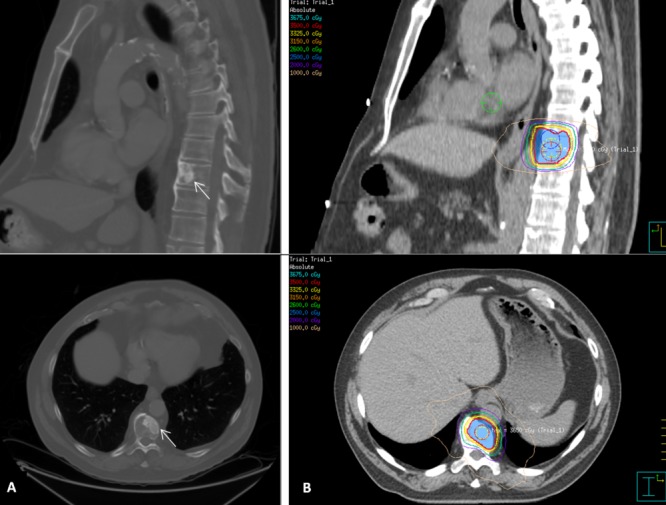
A) Sagittal and axial CT slices showing the T10 oligometastasis. B) Stereotactic body radiotherapy treatment plan and dose distribution.

Given that Mr. X presented with one site of metastatic disease from which he was asymptomatic, management options that were offered included: observation, palliation, and stereotactic body radiotherapy (SBRT). Mr. X ultimately underwent SBRT to a dose of 3500 cGy in five fractions, delivered every other day, to the oligometastasis and completed treatment on August 12, 2013; the treatment plan was created so that at least 95% of the planning target volume would receive at least 95% of the prescribed dose. His imaging and treatment plan is shown in Figure 2. He achieved a complete PSA response within six months. Mr. X remains on a complete androgen blockade, and his PSA was 0.02 at his most recent follow-up in May 2015.

Written consent was obtained from the patient for his treatment and this case report.

## Discussion

Prostate cancer is the second most common cancer in men worldwide and accounted for 14% of all new cancer cases in the United States in 2014 [3]. A subset of men ultimately develops disseminated disease after definitive treatment, demonstrated by an elevated PSA, symptoms, or radiographic changes, and initial therapy commonly consists of androgen deprivation therapy (ADT). Unfortunately, most men on ADT will eventually develop castrate-resistant prostate cancer (CRPC), defined as biochemical, radiographic, or symptomatic progression despite castrate levels of testosterone (< 1.7 nmol/L) [4]. While a number of treatment options exist at this stage, including targeted therapy, immunotherapy, and chemotherapy, this disease is considered incurable and the median overall survival in men with CRPC is approximately four years [5].

Classically, systemic therapy has been considered the standard of care for patients with metastatic cancer. While systemic therapy improves survival in some patients, it is rarely curative in this setting. Emerging evidence suggests that an intermediate oligometastatic state between limited disease and widespread metastases exists; theoretically, with locally ablative treatment to a limited number of metastases, long-term survival may be achieved. The oligometastatic state is defined as those patients who develop a limited number of metastatic deposits and in limited organ sites [6].

Although oligometastases may represent more indolent tumor biology, long-term survival has been reported after aggressive local management of oligometastases from a number of tumor sites and histologies. For example, 10-year survival rates of 17% to 28% have been reported with surgical resection of hepatic metastases from colon cancer; this is improved from patients treated with systemic therapy alone [7]. Similarly, in a systematic review of over 1,000 patients, resection of hepatic metastases from breast cancer was associated with five-year survival rates of 21% to 61% [8]. Five-year survival of 39% to 53.5% has been reported following pulmonary metastasectomy from colorectal cancer, renal cell carcinoma, and gynecologic cancers [9-11]. For patients who are unable to undergo surgical resection, stereotactic body radiotherapy (SBRT) allows for the delivery of ablative doses of radiation to oligometastases and has been shown to offer durable local control in various sites. For example, local control rates for lung and liver metastases from a number of tumor sites range from 70 to 90% at two years [12].

The majority of patients with CRPC ultimately develop distant disease with bone being the most common site of metastatic cancer spread [5]. Bone metastases are commonly associated with pain, morbidity, functional impairment, and a diminished quality of life. Similar to other tumor sites, patients with oligometastases from prostate cancer have a better prognosis compared with patients with extensive metastatic disease and may respond favourably to aggressive management. Ost, et al.performed a systematic review to evaluate metastasis-directed therapy, consisting of either high-dose radiotherapy or surgery, in the setting of oligometastatic prostate cancer recurrence [13]. Radiotherapy was delivered using conventional fractionation, hypofractionation, or SBRT, and surgically treated patients underwent salvage lymph node dissections [13]. While definitions for biochemical recurrence and progression varied in the included studies, approximately half of patients were progression-free one to three years following treatment [13].

Emerging evidence has shown SBRT to be particularly effective in achieving durable local control. While most reported evidence comes from single-institution series with limited numbers of patients, local control rates ranging from 84% to 100% have been reported; prescribed doses in these studies were most commonly 16 Gy to 20 Gy in one fraction, 27 Gy in three fractions, or 30 Gy in five fractions [14].In one series, for example, fifty non-castrate-resistant patients with up to three synchronous oligometastases, who were treated with SBRT to a dose of 30 Gy in three fractions or a dose of 50 Gy in 10 fractions, achieved a local control rate of 100% at a median follow-up of two years [15]. Similarly, Muacevic, et al. delivered a median dose of 20 Gy in one fraction to 40 patients with bony metastases from prostate cancer and reported a two-year local control rate of 95.5% and no Grade 3 or higher toxicities [16]. Ahmad, et al.reported similar outcomes; notably, however, in their study 52.9% (n=9) of patients, three of whom had CRPC, had an undetectable PSA at a median follow-up of three months (range: 1-8 months) [17].

There are several ongoing trials evaluating SBRT in the treatment of oligometastatic disease. SABR-COMET compares SBRT and standard chemotherapy and radiotherapy in patients with good performance status and one to five sites of metastatic disease to assess the impact on overall survival and quality of life [18]. NRG-BR001 is a Phase 1 study designed to determine the optimal SBRT dose for a number of metastatic locations in the setting of oligometastases [19].Lastly, STOMP is a Phase II trial randomizing patients with an oligometastatic recurrence of prostate cancer between metastasis-directed therapy with surgery or SBRT followed by active surveillance versus active surveillance only [20]. The results of these trials will provide valuable information regarding both the efficacy of metastasis-directed therapy in this patient population as well as potential risks and toxicities.

## Conclusions

In conclusion, previous studies have supported aggressive management of oligometastases in prostate cancer, although most have focused on non-castrate patients. Only Ahmad, et al.reported a complete biochemical response in the setting of CRPC; however, the median follow-up in this study was only three months. This case supports the use of SBRT in oligometastatic CRPC and suggests that aggressive management of limited metastases may provide good long-term outcomes in well-selected patients.
